# Combining targeted drugs to overcome and prevent resistance of solid cancers with some stem-like cell features

**DOI:** 10.18632/oncotarget.2424

**Published:** 2014-09-02

**Authors:** Elina Jokinen, Niina Laurila, Peppi Koivunen, Jussi P. Koivunen

**Affiliations:** ^1^ Department of Medical Oncology and Radiotherapy, Oulu University Hospital, University of Oulu, Oulu, Finland; ^2^ Biocenter Oulu, Faculty of Biochemistry and Molecular Medicine and Oulu Center for Cell-Matrix Research, University of Oulu, Oulu, Finland

**Keywords:** Targeted cancer therapy, resistance, cancer stem-like cells, non-small cell lung cancer

## Abstract

Treatment resistance significantly inhibits the efficiency of targeted cancer therapies in drug-sensitive genotypes. In the current work, we studied mechanisms for rapidly occurring, adaptive resistance in targeted therapy-sensitive lung, breast, and melanoma cancer cell lines. The results show that in *ALK* translocated lung cancer lines H3122 and H2228, cells with cancer stem-like cell features characterized by high expression of cancer stem cell markers and/or *in vivo* tumorigenesis can mediate adaptive resistance to oncogene ablative therapy. When pharmacological ablation of *ALK* oncogene was accompanied with PI3K inhibitor or salinomycin therapy, cancer stem-like cell features were reversed which was accompanied with decreased colony formation. Furthermore, co-targeting was able to block the formation of acquired resistance in H3122 line. The results suggest that cells with cancer stem-like cell features can mediate adaptive resistance to targeted therapies. Since these cells follow the stochastic model, concurrent therapy with an oncogene ablating agent and a stem-like cell-targeting drug is needed for maximal therapeutic efficiency.

## INTRODUCTION

The efficiency of targeted cancer therapies in drug-sensitive genotypes such as *HER2* amplified breast cancer, *B-Raf* mutant melanoma and *ALK* translocated non-small cell lung cancer (NSCLC) is significantly inhibited by treatment resistance. This resistance is often divided into early *de novo* resistance, in which cancer cells are initially unaffected by the drug, and late, acquired resistance, in which the cells gain resistance by a mechanism that abolishes the effect of the drug. Furthermore, adaptive resistance mechanisms also occur in which cells are able to survive in the presence of the drug, remaining in either a dormant or a slowly dividing state.

Tumor heterogeneity is often explained by cancer stem cell (CSC) models. In these hierarchic models, cells having CSC potential and ability to generate cells with self-limited proliferative capacity maintain the CSCs pool. Furthermore, clonal evolution of cells with additional genetic alterations is another driving force for tumor heterogeneity. These genetic alterations can produce cells with self-renewing and proliferating capacity resulting generation of cancer stem-like cells (CSLC) [1-3].

High tumorigenity in xenograft models is taken as the gold standard for the identification of CSCs or CSLCs, but they can also be identified by various cell surface markers such as CD44high/CD42low (breast cancer), CD133high (glioblastoma) and high aldehyde dehydrogenase 1 (ALDH1) expression or activity (various solid tumors) [4-7]. Epithelial-to-mesenchymal transition (EMT) has been linked to the cancer stem cell phenotype in many studies [8, 9]. Presence of cells with CSC features has been connected with a poor patient outcome [4, 10] and with resistance to traditional chemotherapy and radiotherapy [11, 12]. Some works have also shown association of these markers to targeted therapy resistance [13, 14]. Studies have shown that traditional cancer therapies preferentially target the proliferating, differentiated cells rather than the CSCs, although some pharmacological agents such as salinomycin, abamectin, etoposide, and disulfiram have been shown to target CSLCs *in vitro* [15-17]. Furthermore, various signalling pathways have been linked to the cancer stem cell phenotype *e. g.* Wnt, Notch and ß-catenin [18].

The acquired resistance to targeted therapies that affects all patients with metastatic disease can occur through various mechanisms, such as point mutations in the target gene that lower its affinity for the drug, activation of other tyrosine kinases, and EMT [19]. The role of adaptive resistance and CSLSs in acquired resistance to targeted therapies remains largely unexplored. Cancer cells capable of undergoing adaptive resistance could be responsible for the minimal residual disease and serve as a source of acquired resistance.

The current study investigates the role of cells with CSLC features in resistance to targeted cancer therapies for NSCLC, breast cancer and melanoma. Furthermore, it considers drug combinations capable of inhibiting cells with CSLC features in adaptive, and acquired resistance.

## RESULTS

### Adaptive resistance to ALK inhibition is mediated by ALHD1-positive cells

H3122, an *ALK*–translocated and dependent NSCLC cell line, was selected as an initial model for studying adaptive drug resistance. Pharmacological inhibition of ALK in H3122 cells causes marked cell death, although a small fraction of the cells do survive for long periods of time but without marked proliferation. When the ALK inhibitor is withdrawn, the cells rapidly start to proliferate, and when re-challenged with the drug, a marked response is seen once more. Furthermore, H3122 forms papillary structures *in vitro*, which are abolished by treatment with ALK tyrosine kinase inhibitor (TKI), suggesting some form of hierarchy among the tumor cells. We thus hypothesized that this would be a good model for studying the mechanisms of adaptive resistance to targeted cancer therapy.

**Figure 1:**
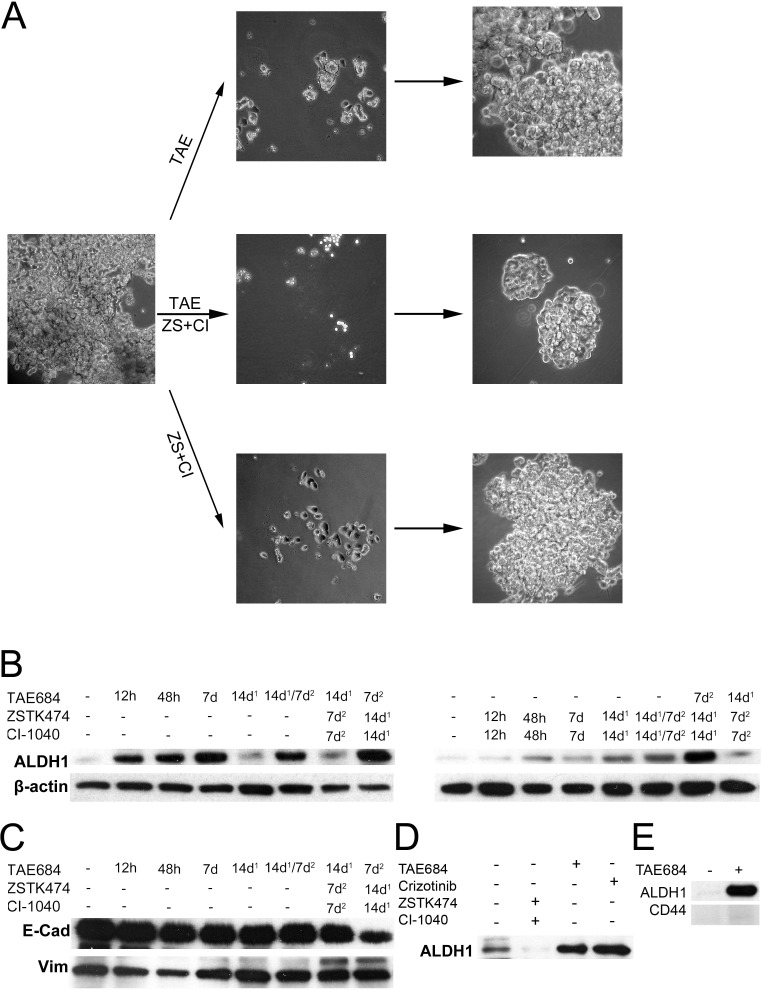
ALK inhibitor and ALDH1 in the H3122 NSCLC line H3122, an *ALK*-translocated cell line, was exposed to ALK TKI (TAE) or a combination of PI3K and MEK inhibitors (ZS+CI) or both. (A) phase contrast micrograph of the cells when exposed to the drugs for 7d, after which they were allowed to repopulate. (B) Western blot analysis for ALDH1 in H3122 cells treated with the same drugs for the times indicated. 14d^1^ signifies that the cells were exposed to the drug for 7d, after which they were allowed to recover for an additional 14d, while 14d^1^/7d^2^ denotes exposure to a specific drug first for 7d followed by 14d recovery and then another 7d exposure. (C) E-cadherin (E-Cad) and vimentin (Vim) expression in the same samples as in panel B. (D), ALDH1 expression in H3122 cells treated with ZSTK474+CI-1040, TAE684 or crizotinib for 7d. (E), Expression of ALDH1 and CD44 after TAE684 exposure in H3122 cells. TAE684 was used at a concentration of 0.1μM, ZSTK474 at 3.3μM, CI-1040 at 1μM and crizotinib at 1μM.

We first treated H3122 cells with the ALK inhibitor TAE684 for 7 days, after which the drug was withdrawn and the cultures were analyzed by phase contrast microscopy. As in previous studies, TAE684 induced marked cell death, a decline in cell numbers and a disappearance of the papillary structure. When TAE684 was withdrawn, the remaining cells quickly started to re-proliferate and form papillary structures (Figure 1A). After 14 days of re-growth, they were re-challenged with TAE684, eliciting a similar response to that following the initial drug challenge (not shown). H3122 cells are also highly sensitive pharmacologically to concurrent dual inhibition of PI3K and MEK kinases [20, 21]. As with ALK inhibition, dual inhibition of PI3K and MEK induced marked cell death and a reduction in the number of cells during 7 days of treatment followed by a rapid recovery of the cell population after drug withdrawal (Figure 1A). Furthermore, a re-challenge with the same drug regimen resulted in a similar response as initially (not shown).

Next we wanted to investigate whether H3122 cells respond differently to a combination of ALK treatment and dual PI3K/MEK inhibitor treatment. The responses and re-growth occurred in the same manner regardless of whether the cells were first challenged with the ALK inhibitor and after regrowth with dual PI3K and MEK inhibition or *vice versa* (not shown). Conversely, the magnitude of the rate of repopulation was markedly reduced, but not blocked, when both drug regimens were administered concurrently (Figure 1A).

We speculated that cells showing adaptive resistance might bear a CSLC phenotype, and we therefore studied the expression of ALDH1, a marker of CSCs, in the same experimental setting using Western blot analysis. ALHD1 expression was low in untreated H3122 cells, but the ALK inhibitor treatment with TAE684 induced it gradually but to a marked extent from 12 h of treatment onwards (Figure 1B). A similar increase in ALDH1 was also seen with crizotinib, another unrelated ALK inhibitor, suggesting that the phenomenon is related to ALK inhibition rather than to any specific inhibitor (Figure 1D). When TAE684 was withdrawn for 14 days, ALDH1 expression remained at the initial, low level. When the cells were re-challenged after regrowth with TAE684 similar induction of ALHD1 was detected. When the cells were initially challenged with TAE684 and later with dual PI3K/MEK inhibition, no marked increase in ALDH1 expression was detected, while reversing the order produced a marked increase in ALDH1 expression. When the H3122 cells were challenged initially with dual PI3K/MEK inhibition, a low level of induction of ALDH1 expression was detected, which did not disappear after drug withdrawal (Figure 1B).

We next wanted to investigate whether any other CSC marker correlated with ALDH1 in the H3122 line. Since EMT has been linked to cancer stem cells, we set out to study the EMT markers E-cadherin and vimentin in this context. We could not see any downregulation of E-cadherin or upregulation of vimentin, however (Figure 1C). Expression of CD44, a marker linked to the CSC phenotype in some cancers, was also undetectable in the H3122 cells both before and after ALK inhibition (Figure 1E).

### TAE684-Treated, ALHD1-positive cells are highly tumorigenic in the xenograft model

**Figure 2:**
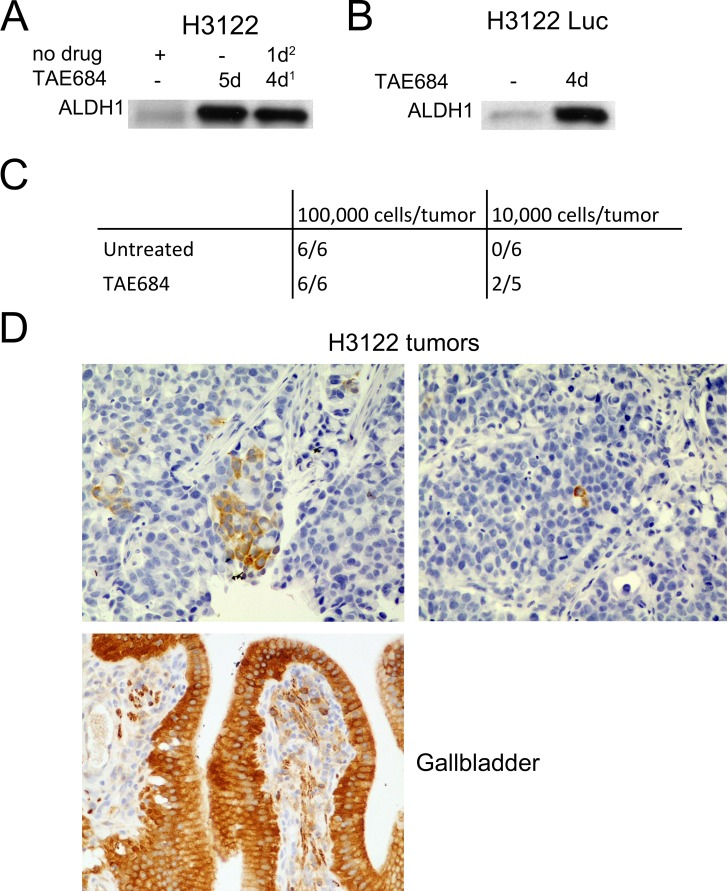
In *Vivo* Tumorigenesis of TAE684-Pretreated Cells (A) Western blot analysis for ALDH1 expression in H3122 cells treated with TAE684 for 5d or 4d followed by 1d recovery. (B) ALDH1 expression in H3122 Luc cells after 5d exposure to TAE684. (C) number of tumors per animal in mice injected with 100,000 or 10,000 H3122 Luc cells and either untreated or pretreated with TAE684. TAE684 treatment was continued for 14d, after which the cells were allowed to recover for 1d before injection into the mice. (D) ALDH1 expression in H3122 xenograft tumors and in human gall bladder.

H3122 cells transfected with a luciferase reporter gene were shown to have a similar level of ALDH1 induction and cytotoxic response to TAE684 *in vitro* as the original, untransfected cell line. Furthermore, cells treated for 4 days with TAE684 following drug withdrawal of 24 h before analysis, showed the induced ALDH1 expression (Figure 2A-B). Next, the H3122 Luc cells were treated with TAE684 for 14 days to induce ALDH1-positivity and the drug was withdrawn 24 h before injection of the cells into mice, to diminish the potential negative effects of the drug on tumorigenesis. 10,000 or 100,000 of either non-treated control or TAE684-treated, luciferase reporter-incorporated H3122 cells were injected subcutaneously into mice on both flanks. The number injected cells was selected based on preliminary experiments with H3122 in which 100,000 cells was able to generate tumour in most mice while no tumour formation was seen 10,000 or 1,000 cells injected. All the mice injected with 100,000 cells in both groups rapidly generated tumors, with a similar rate of occurrence, but an increase in tumorigenesis was seen in the mice injected with 10,000 cells in the tumors initiating from TAE684-treated cells, so that 40% of these mice developed tumors whereas none of the mice injected with control cells had tumors during the follow-up period of 9 weeks (Figure 2C).

Dissected tumors (100,000 cells) from both non-treated and TAE684-treated H3122 cells were analyzed for immunohistochemical ALDH1 expression. Human gallbladder epithelium, in which a high expression of ALDH1 has been reported in previous studies, served as a positive control. Patchy expression of ALDH1 was detected in the H3122-derived tumors, with only a very few positive tumor cells, arranged either in small groups or as single cells (Figure 2E). We could not detect any major difference in the number or arrangement of ALDH1-positive cells in either group of tumors (not shown).

### Pharmacological Screening for an Agent Targeting ALDH1-Positive Cells

**Figure 3:**
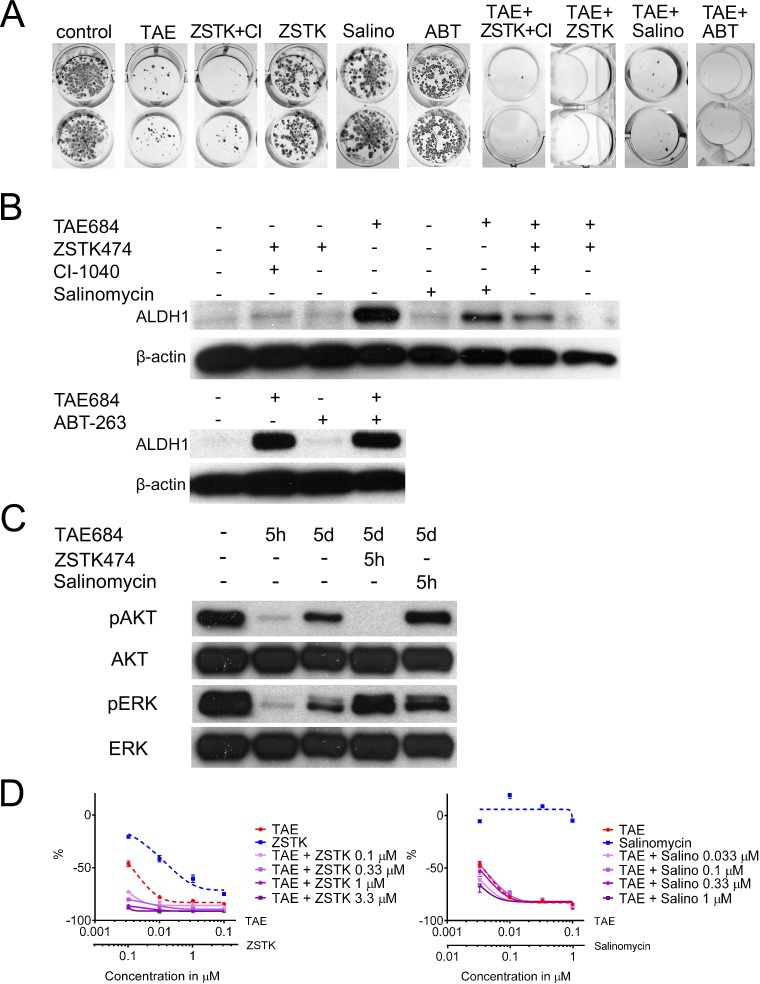
Drug Combinations for Promoting Adaptive Resistance and the Cancer Stem-Like Cell Phenotype (A) Colony formation assay of H3122 cells treated with TAE684 (TAE), ZSTK474+CI-1040 (ZSTK+CI), ZSTK474 (ZSTK), Salinomycin (Salino) or ABT-263 (ABT) or their combinations for 7d, after which the colonies were allowed to regrow. (B) Western blot analysis for ALDH1 in H3122 cells after 5d treatments with the same drugs or their combinations. (C) Western blot analysis for phosphorylated and total AKT and ERK with the drug treatments and exposures times indicated. (D) 72h MTS cytoxicity assay of H3122 cells treated with TAE684, ZSTK474, Salinomycin or their combinations. The single agent treatments are presented with a dashed line and the combination treatments with a continuous line. The Y-axis indicates the percentage decrease in viability relative to untreated cells. The drug concentrations used in the experiment, unless otherwise indicated, were TAE684 0.1μM, ZSTK474 3.3μM, CI-1040 1μM, salinomycin 0.1μM and ABT-263 1μM.

The next stage was to investigate whether a combination of pharmacological agents could target cells with adaptive TKI resistance *in vitro* using the H3122 colony formation model. H3122 cells were treated with TAE684 in combination with another pharmacological agent (Table 1) for 7 days, after which the drugs were withdrawn. After regrowth had occurred, the colonies were fixed and stained. The pharmacological agents were small molecular inhibitors in use or under investigation as cancer therapeutics, drugs known to target stem cells or pathways involved in these, or traditional chemotherapeutic agents. Surprisingly, only three agents, the PI3K inhibitor ZSTK474, the BH3 mimetic ABT-263 and the cancer stem cell-specific drug salinomycin, were able to inhibit colony formation in combination with TAE684. ZSTK474 possessed this activity as a single agent in the H3122 line, whereas ABT-263 and salinomycin had no activity alone (Figure 3A). Initial phase contrast analysis had also suggested that dual PI3K/MEK inhibition would reduce colony formation with TAE684, but the pharmacological screening showed that only PI3K inhibition is needed for such inhibition and that the MEK inhibitor alone or in combination with TAE684 did not have any activity in this setting (Figure 3A). PI-103, a PI3K/mTOR inhibitor, also induced a similar inhibition of colony formation alone and in combination with TAE684 to that observed with ZSTK474, suggesting that it is a PI3K inhibition-mediated class effect (not shown).

**Table 1:** Compounds Used in the Drug Screen for Adaptive Resistance in H3122 line

Compound	Target/Class	Concentration in μM
Erlotinib	EGFR	1
Lapatinib	HER2	1
Crizotinib	ALK/MET	1
TAE684	ALK	0,1
AG1024	IGF1R1	10
Sunitinib	Multi-TKI	1
Dasatinib	Multi-TKI	1
Sorafenib	Multi-TKI	1
CI-1040	MEK	1
ZSTK474	PI3K	3,3
PI-103	PI3K/mTOR	1
Gö6976	PKC	1
Vemurafenib	B-Raf V600E	1
Salinomycin	Stem Cells	1
Abamectin	Stem Cells	1
Disulfiram	Disulfiram	0.01, 0.1, 1
Ly-2157299	TGF-ßR1	1
SD 208	TGF-ßR1	1
NVP-XAV939	WNT	1
DBC	γ-Secretase	1
Vismodegib	Hedgehog	10
Entinostat	HDAC	1
5-AZA	Methylation	1
Chloroquine	Autophage	10
ABT-263	BH3	1
Etoposide	Chemo	1
Cisplatin	Chemo	1
Paclitaxel	Chemo	0.1

Since ZSTK474, ABT-263 and salinomycin inhibited colony formation in combination with TAE684, we then investigated the effect of these combinations on ALDH1 expression. When the H3122 cells were treated with salinomycin, ZSTK474, or ZSTK474 with CI-1040 (MEK inhibitor) no marked increase in ALDH1 expression was seen, while TAE684 induced high ALDH1 expression. When TAE684 was combined with salinomycin, ZSTK474, or ZSTK474 and CI-1040, marked inhibition of ALDH1 induction was seen, this being most prominent in the regimens containing the PI3K inhibitor. ABT-263 treatment alone or in combination with TAE684 did not alter the ALDH1 expression (Figure 3B), and therefore we focused our further experiments entirely on the ZSTK474 and salinomycin combinations.

We then set out to investigate how AKT and ERK1/2 signalling activity, the immediate downstream effectors of PI3K and MEK, alter in H3122 cells treated with TAE684 according to different time schedules. A 5-hour treatment with TAE684 induced a striking downregulation of phosphorylated AKT and ERK1/2, but when the treatment was continued for 5 days the phosphorylation of AKT and ERK1/2 increased again. The re-activation of AKT could be abolished by a 5-h treatment with ZSTK474 (Figure 3C). When the H3122 cells were treated with salinomycin for 5 days, no change in either AKT or ERK was detected, suggesting that salinomycin inhibits ALDH1-positive cells downstream of AKT signalling (Figure 3C).

In order to analyze further whether combinations of the ALK inhibitor with the PI3K inhibitor or salinomycin behave similarly in short-term assays as they had done in the longer-exposure experiments such as the colony formation assay, H3122 cells were exposed to TAE684 with or without increasing concentrations of ZSTK474 or salinomycin for 72 h and analyzed with a MTS cytotoxicity assay. The TAE684+ZSTK474 combination had a synergistic effect in the short-term assay as well, but the combining of salinomycin with TAE684 did not alter the toxicity of the therapy (Figure 3D).

### Cancer stem cell markers in targeted therapy-sensitive cell line models

**Figure 4:**
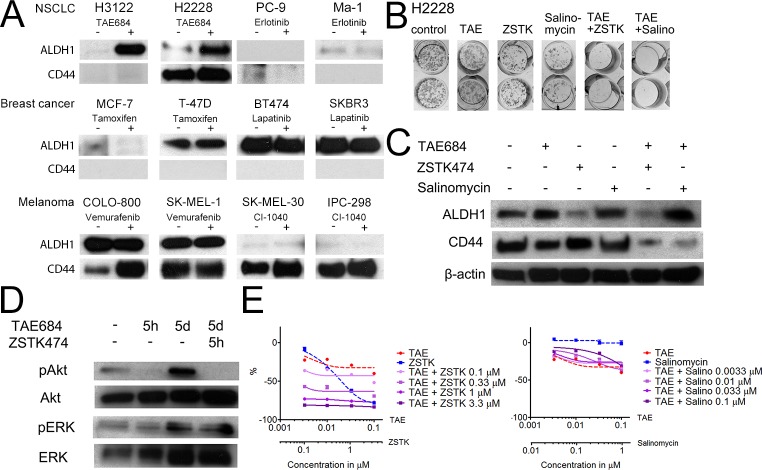
Stem Cell Markers in the Targeted Therapy-Sensitive Cell Lines (A) Western blot analysis for ALDH1 and CD44 in the cell lines indicated after 7d exposure to the targeted drugs. (B) Colony formation assay of H2228 exposure to TAE684 (TAE), ZSTK474 (ZSTK), salinomycin (Salino) or their combinations for 7d, after which the colonies were allowed to repopulate. (C) Western blot analysis for ALDH1 and CD44 expression in the H2228 line after 5d exposure to the drugs or combinations indicated. (D) Western blot analysis for phosphorylated AKT and ERK1/2 or their corresponding total proteins in H2228 cells after the drug treatments or their combinations and the exposure times indicated. (E) 72h MTS cytoxicity assay of H2228 cells treated with TAE684, ZSTK474, salinomycin, or their combinations. Single agent treatments are presented with a dashed line and combination treatments with a continuous line. The Y-axis indicates the percentage decrease in viability relative to untreated cells. The drug concentrations used in the experiments, unless otherwise indicated, were TAE684 0.1μM, erlotinib 1μM, tamoxifen 1μM, lapatinib 1μM, vemurafenib 1μM, CI-1040 1μM, ZSTK474 3.3μM and salinomycin 0.1μM.

Since TKI treatment of the *ALK*-translocated NSCLC H3122 cells induced high expression of ALDH1 *in vitro* and increased tumorigenesis *in vivo*, it was necessary to ascertain whether this is a general phenomenon in targeted therapy-sensitive cancer cell lines. One *ALK* translocated (H2228), two *EGFR* mutant NSCLC lines (PC-9, Ma-1), two *HER2*-amplified (BT-474, SKBR-3) and two ER+ breast cancer lines (MCF-7, T-47D), two *B-Raf* mutant (COLO-800, SK-MEL-1) and two *N-Ras* mutant melanoma lines (SK-MEL-30, IPC-289) were exposed to TAE684, erlotinib, lapatinib, tamoxifen, vemurafenib and CI-1040, respectively, for 7 days and analyzed for the expression of ALDH1 and CD44. High endogenous ALDH1 expression was detected in the *HER2*-amplified and T-47D breast cancer lines and in the *B-Raf* mutant melanoma lines. H2228 was the only other line to H3122 cells to display any increased ALDH1 expression in response to targeted therapy. CD44 expression was seen in H2228, all the melanoma lines, with some increase in expression in SK-MEL-30 and COLO-800 in response to treatment (Figure 4A).

As the H2228 line increased expression of ALDH1 in response to TKI, we further examined whether this cell line would behave like the H3122 cells in response to combined inhibition of ALK with PI3K, MEK or salinomycin. Single agents had very limited or no activity on the colony formation while the combination of TAE684 with ZSTK474 or salinomycin induced marked inhibition of colony formation (Figure 4B). We further studied whether the expression of ALDH1 or CD44 was altered by the combination therapies. In H2228 cells, a decrease in ALDH1 expression was seen with ZSTK474 and TAE684+ZSTK474 but not with salinomycin combinations (Figure 4C). Conversely, CD44 expression remained unaltered with single agents but markedly downregulated in response TAE684 and ZSTK474 or salinomycin. We were also anxious to find out whether cell signalling is altered in the H2228 line in response to short or long exposures to TAE684. 5 hours of treatment with TAE684 resulted in complete inhibition of pAKT signalling, while pERK remained unchanged, but by 5 days marked upregulation of pAKT and pERK/ERK was detected even though the cells had been subject to continuous TAE684 treatment. When the cells treated with TAE684 for 5 days were exposed to ZSTK474 for 5 h, complete downregulation of pAKT was seen (Figure 4D).

As with H3122 cells, H2228 cells were exposed to drugs in a MTS cytotoxicity assay in order to analyze further whether combinations of the ALK inhibitor and the PI3K inhibitor or salinomycin behave similarly in short-term assays as they had done in the longer-exposure colony formation experiments. TAE684 and salinomycin had no or limited activity by themselves while ZSTK474 showed concentration dependent cytotoxicity. The TAE684+ZSTK474 combination had a synergistic effect in the short-term assay as well, but the combination of salinomycin with TAE684 did not alter the toxicity of the therapy (Figure 4D).

### Adaptive resistance as a factor in the development of acquired resistance

**Figure 5:**
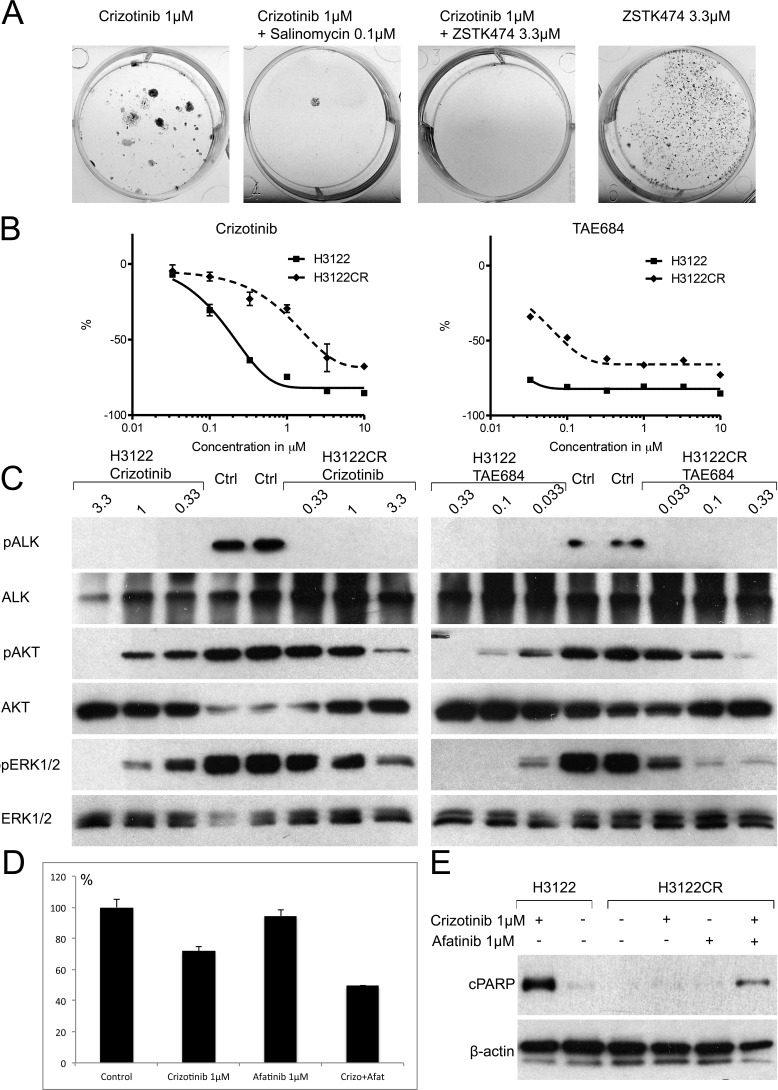
Acquired Resistance in the H3122 Cell Line (A) Fixed and stained plates of H3122 cells were exposed to the drugs or drug combinations indicated for 3mo. (B) 72h MTS cytotoxicity assay of the H3122 or H3122CR cell lines with crizotinib or TAE684. The Y-axis indicates the percentage decrease in viability relative to untreated cells. (C) Western transfer analysis for phosphorylated ALK, AKT, ERK1/2 or their corresponding total proteins in the H3122 or H3122CR cell lines after 5h exposure to the drugs indicated. (D) MTS cytotoxicity assay of H3122CR cells exposed to crizotinib, afatinib or their combination for 72h. The Y-axis indicates the percentage change in viability relative to untreated cells. (E) Western blot analysis for the apoptosis marker cleaved PARP in H3122 or H3122CR cell lines exposed to the drugs or drug combinations indicated for 24h.

Since our data suggested that the PI3K inhibitor and salinomycin can inhibit adaptive resistance in H3122 cells, we asked whether adaptive resistance could serve as source of acquired resistance. Long-term ALK TKI treatment of the H3122 cells has been linked to the development of acquired resistance by multiple mechanisms such as the L1196M secondary mutation and the activation of EGFR/HER2 signalling [22-24]. We therefore decided to expose H3122 cells to 1μM crizotinib with or without 3.3μM ZSTK474 or 0.1μM salinomycin, concentrations previously shown to inhibit adaptive resistance, and to monitor the cells for the development of acquired resistance. After a three-month exposure to the drugs, growing colonies were seen amongst the crizotinib-treated cells (Figure 5A), but visual assessment suggested that the proliferation rate was lower than in the original cell line. Conversely, there were no growing colonies present in either the crizotinib+ZSTK474-treated plates nor the crizotinib+salinomycin-treated ones (Figure 5A). We then monitored the combination-treated cells for an additional three months, but no growing colonies or living cells remained at the end of the treatment period, and therefore no further experiments were carried out with this material.

The resistance mechanism of crizotinib-treated H3122 cells (H3122CR) was further studied by means of cytotoxicity and Western blot assays. H3122CR showed a ~10 fold increase in IC50 concentrations in the MTS cytotoxicity assays, in response to both crizotinib and TAE684 as compared with the original line. Furthermore, the percentage downregulation relative to the controls was markedly lower in the H3122CR cells with both drugs, suggesting an increased number of living cells even at high drug concentrations (Figure 5B). We next exposed the H3122 and H3122CR cells to increasing concentrations of crizotinib and TAE684 for 5 h and analyzed the cells for the phosphorylation of ALK and its downstream targets AKT and ERK. In both cell lines the lowest concentration of the both drugs was able to completely block ALK phosphorylation. Downregulation of AKT and ERK phosphorylation was seen in both cell lines in response to either crizotinib or TAE684, but complete blocking of the signal was seen only in the H3122 cells, suggesting that the resistance is mediated by the presence of another activated receptor, tyrosine kinase (RTK) (Figure 5C). Since EGFR and HER2 have been linked to ALK TKI resistance, we then set out to see whether these RTKs could be mediators of acquired resistance in H3122CR cells. A Western blot assay did not show any increase in either phosphorylated or total EGFR or HER2 levels in the H3122CR cells relative to the control cells (not shown), but when the cells were exposed to afatinib, a dual EGFR and HER2 inhibitor, in combination with crizotinib, markedly increased cytotoxicity was evident by comparison with the single agent treatments. Furthermore, cPARP expression, an indicator of apoptosis, was only present in H3122CR cells when they were exposed to both drugs, but not with single agent treatments (Figure 5D).

We also tried to generate colonies of H3122CR cells to see whether multiple resistance mechanisms occurred simultaneously in the cell line. However, the H3122CR lost their acquired resistance, as analyzed by either cytoxicity or Western blot assays (not shown), during the colony generation phase. We next withdrew the H3122CR cells from the crizotinib medium and analyzed them for the presence of crizotinib resistance by means of cytotoxicity assays. The cells had retained their resistance after three and six passages of drug withdrawal, but resistance had been abolished after nine passages (not shown).

## DISCUSSION

Acquired drug resistance in targeted therapy sensitive cancers has been a topic of extensive studies. In solid malignancies, clinical manifestation of acquired resistance occurs from months to some years after initiation of the therapy. Adaptive resistance mechanism, which occur rapidly after therapy initiation, is another mechanism how cancer cells can avoid targeted drug induced cell death. Even though adaptive resistance is largely accepted as a mechanism for targeted drug resistance, it remains largely an unexplored area. Current work sheds light on the occurrence, potential mechanisms, and drug combinations overcoming this form of resistance using *in vitro* models.

We investigated adaptive resistance to targeted cancer therapies with the main aim of ascertaining whether the cells with CSLS features could play a role in such resistance. Our findings indicate that increased expression of CSC markers and *in vivo* tumorigenesis may occur in response to targeted therapies at least in some cell lines. We cannot conclude how general this phenomenon is, since only two out of the twelve cell lines studied here presented the same phenomenon. Even though multiple markers such as CD44, CD133, ALDH1 and EMT have been linked to the CSLC phenotype, no general marker for CSCs has been identified [1, 3]. Consequently, the CSLC phenotype could exist even though we did not see any change in the markers that we studied. As in previous works, our study also showed some discordancy between ALDH1 and CD44 markers in the H2228 line [1]. Based on our work, it remains unproven whether the cells with CSLC features exist as a small subpopulation before initiation of drug treatment or if CSLC markers are upregulated in response to drug treatment.

Our screening for an agent targeting adaptive resistance in H3122 cells, an ALK-translocated NSCLC line, included 28 selected agents which affected the general signalling pathways, CSLSs, or cytotoxics. Only three agents were found to inhibit adaptive resistance, including a PI3K inhibitor, the CSLC-targeting drug salinomycin and the BH3-mimetic. ZSTK474 and salinomycin also inhibited expression of the CSC marker ALDH1, suggesting that cells with CSLS phenotype could be mediators of adaptive resistance. PI3K inhibitors alone also possessed cytotoxic activity in H3122 cells in short-term assays, so that we cannot completely rule out the possibility that PI3K activity with respect to adaptive resistance might be partly mediated by increased acute cytotoxicity rather than stem cell targeting. Salinomycin, however, is non-cytotoxic in itself, implying that it may play a role as a CSC targeting agent. Salinomycin was originally discovered as a CSLS targeting agent in a ~16,000-compound chemical screen which showed it to be the most selective agent against CSLCs [15]. Previous works have also linked PI3K-AKT-mTOR signalling with the CSLC phenotype [25, 26]. In the H3122 and H2228 cells PI3K-AKT signalling activity increased in long-term (days) ALK TKI exposures after initial downregulation (hours). H2228, an ALK translocated NSCLC line, has been shown to have diminished activity with primary *ALK* TKIs through unknown mechanism [27]. Current work suggests that CSLC features might be related to the primary ALK TKI resistance in H2228 line, which has basal expression of both ALDH1 and CD44 and downregulation of latter correlates with cytotoxicity.

Acquired resistance is an important factor limiting the efficiency of targeted therapies, and one that occurs in virtually all patients with metastatic disease in the course of time. It can occur through various mechanisms such as point mutations in the target gene, the activation of other tyrosine kinases, and EMT [19]. The dominant theory regarding the development of acquired resistance is simply Darwinian selection among the drug-resistant clones initially present in the tumor. Interestingly, a recent work concerned with vemurafenib resistance has provided evidence for a knottier background in acquired resistance. In this model, drug-dependent clones can mediate resistance and discontinuous dosing of the drug can forestall this resistance [28]. Our work also provides evidence for a more complex development of acquired resistance, since combining drugs that affect cells with CSLC features in combination with ALK TKI in the H3122 cells blocked the development of EGFR and/or HER2 mediated acquired resistance, as has been described previously in the line [23, 24]. This resistance was reversible in nature, as characterized previously [23, 24], and therefore we cannot rule out a continuum of adaptive resistance. However, as we followed the cultures over time, crizotinib-treated cells remained dormant until marked proliferation started to occur after three months of drug exposure. It is possible that resistant clones become diluted after drug removal because of the slower proliferation rate and the diminishing of resistance over time. Diminished acquired resistance has also been shown to occur in genetic alteration-based acquired resistance models such as T790M in *EGFR*-mutant NSCLC [29]. Since the salinomycin-ALK TKI combination blocked the formation of acquired resistance in the H3122 model, it is possible that cells with CSLC features can serve as a source for this form of resistance. PI3K-AKT-mTOR signalling pathway re-activation is very frequently seen in acquired resistance, making it possible that the PI3K-ALK inhibitor combination might prevent acquired resistance directly rather than through cells with CSLC features.

**Figure 6:**
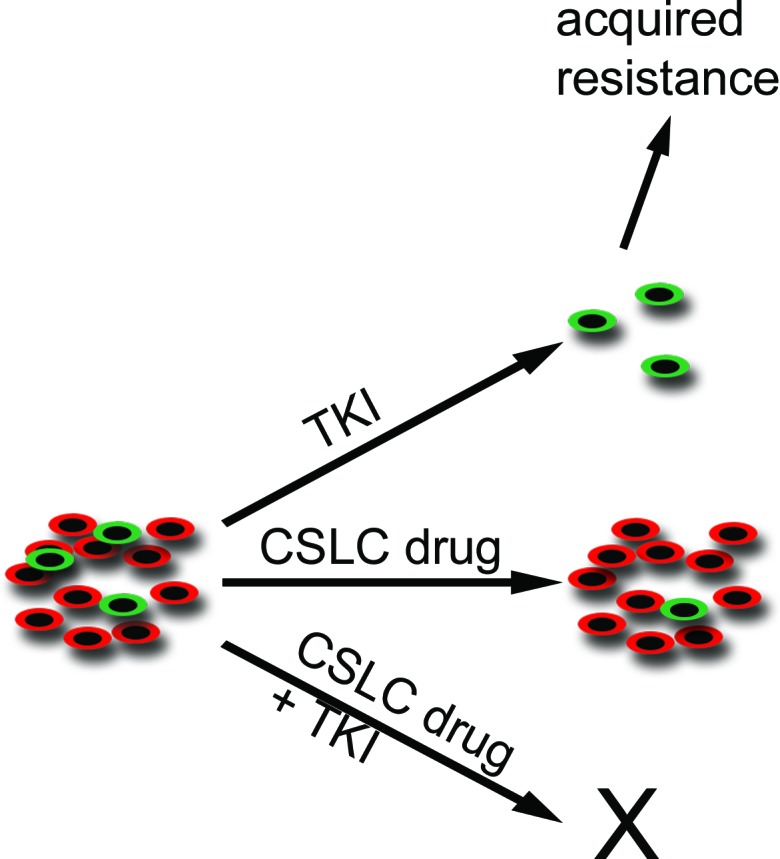
Schematic Representation of Cancer Stem-Like Cells and their Targeting with TKI and Stem-Like Cell Drugs TKI-sensitive malignant tumors consist of differentiated cells (red) and a minor population of cancer stem-like cells (green). When the tumors are challenged with TKI, the major tumor response is seen in the preferred target, the differentiated cells, while the stem-like cells remain unaffected. It is these remaining stem-like cells that may be responsible for tumor relapse and acquired resistance. When the tumor is targeted with a stem-like cell-specific (CSLC) drug, a limited tumor response is seen because of the stochastic nature of the cancer stem cell model. The most prominent response is seen when tumors are targeted concurrently with TKI and a CSLC drug.

Traditional CSC models describe a hierarchic organisation of tumors. Based on hierarchic models, only CSC targeting would be needed for tumor regression [1-3]. There is also evidence that stochastic CSC models which presuppose that tumor cells are plastic with high rate of bi-directional interconversion between stem and differentiated states [30]. If the stochastic model stands, targeting both CSCs and differentiated is needed for maximal therapeutic efficiency. The results of our experiments for adaptive resistance in cells with CSLC features reflect similar stochastic model. In H3122 line, upregulation of CSC marker ALDH1 by ALK TKI was seen also after cytotoxic treatment of cells with dual PI3K and MEK therapy. More importantly, combinatory drug treatment experiments in H3122 and H2228 lines showed that CSLC targeting agents had no or limited single agent activity while their combination with TKI, preferentially targeting non-CSLCs, increased cytotoxicity (Figure 6).

In conclusion, the current work describes adaptive resistance to targeted cancer therapies. It also characterizes drug combinations targeting this form of resistance, and provides evidence that targeting adaptive resistance can prevent the later occurrence of acquired resistance. An understanding of the mechanisms behind resistance can provide clues for smarter therapeutic approaches, which can in turn increase the efficiency of cancer therapies.

## MATERIALS AND METHODS

### Cell lines and inhibitors

The following cell lines were used: NSCLC lines H2228, H3122, and PC-9; breast cancer lines BT474, SKBR3, MCF-7, and T-47D; melanoma lines IPC-298, SK-MEL30, SK-MEL1, and COLO-800. The NSCLC cell lines were kind gifts from Dr. Pasi Jänne (Dana-Farber Cancer Institute, Boston, USA), breast cancer lines were from one of us (P.K.) and the melanoma cell lines were purchased DSMZ GmbH (Braunschweig, Germany). The H3122 Luc cell line was generated by a retroviral transfection with a firefly luciferase plasmid (MSCV-Luc-Puro). All cell lines were grown in RPMI 1640 medium with 10% fetal bovine serum (FBS), 100 units/ml penicillin and 100 μg/ml streptomycin. Cells were incubated at 37°C in a 5% CO_2_ atmosphere. Drugs used with their final concentrations are listed in the supplementary Table 1. All drugs were dissolved in DMSO in the concentration of 10 mM and stored in aliquots at −20°C. Further dilutions were made in the cell culture medium.

### Western blot analysis

The cells were plated on 6-well plates, allowed to attach for 1-2 days, and then treated with drugs. After desired drug treatments, the cells were lysed with NP40 lysis buffer (1% Igepal CA-630, 20 mM Tris-HCl pH 8.0, 137 mM NaCl, 10% glycerol, 2 mM EDTA, 1 mM sodium orthovanadate, 10 μg/ml aprotinin and 10 μg/ml leupeptin). Protein concentrations of the cell lysates were measured using the Bio-Rad Protein Assay (Bio-Rad; Hercules, CA). After equalizing the protein concentrations of the samples, 3× Laemmli buffer added, samples were boiled and stored at −80°C.

Equal amounts of protein samples were separated on SDS-PAGE and proteins were transferred to PVDF membrane. The membranes were blocked and then incubated in primary antibodies overnight at 4°C. Horseradish peroxidase (HRP)-linked antibody was used as a secondary, the membranes were developed using chemiluminescense and exposed on radiographic films. All western blot experiments were performed in duplicates.

The following antibodies were used: ALDH1 (BD Transduction Laboratories, Franklin Lakes, USA), CD44, phospho-Akt (S473), Akt, phospho-ERK1/2 (T202/Y204), ERK1/2, phospho-ALK (T1096), ALK, vimentin, E-cadherin, and Anti-Mouse/Rabbit HRP-linked antibody (Cell Signaling Technologies, Danvers, USA).

### Colony formation assay

300-1300 cells were plated on 24-well plates and treated with drugs for 7 days in multible parallel wells. After 7 days, the drugs were withdrawn and the cells were allowed to proliferate and form colonies for several weeks. After differences in the growth of colonies had appeared, the cells were fixed with methanol and dyed with crystal violet stain.

### MTS cytotoxicity assay

In the MTS assay, 5,000-12,000 cells were plated into 96-well plates and treated with drugs and their combinations for 72 hours. Three to six parallel wells for each treatment were used and untreated cells were used as a control. After the drug treatments, the cells were incubated in MTS-reagent (Promega; Madison, WI) supplemented with phenazine methosulfate (Sigma-Aldrich; St. Louis, MO). The absorbances at 490 nm were recorded on a plate reader (Anthos Reader 2001, Anthos Labtec Instruments, Austria). The results were displayed graphically using the GraphPad Prism software (GraphPad Software; La Jolla, CA) and the curves were fitted using a non-linear regression model with a sigmoidal dose response.

### Mouse xenograft model

H3122 Luc cells were treated with 0.1μM TAE684 for 14 days and let to recover for 1 day before injection. TAE-untreated H3122 Luc cells were used as controls. After trypsination, the cells were counted using trypan blue exclusion, and the cells were diluted with PBS to 100,000 or 10,000 cells/0.2ml and immediately injected on mice. Female NOD.Cg-Prkdc(scid)Hr(hr)/NCrHsd mice (Harlan Laboratories; Boxmeer, Netherlands) were anesthetized with fentanyl/fluanisone/midazolam and 0.2ml of the cell solution was injected on both flanks. Mice were anesthetized with isoflurane, injected intraperitoneally with 15 mg/kg D-luciferin Firefly and imaged weekly using the IVIS Spectrum *in vivo* imaging system with Living Image software (PerkinElmer; Waltham, USA). The luminescent signal was measured as radiance (p/sec/cm2/sr). The animal experiments were performed according to protocols approved by the Provincial State Office of Southern Finland (license number ESAVI-5307-04.10.03-2011).

### Generation of acquired resistance

H3122 cells were plated on 6-well plates, three replicate wells per treatment. The cells were exposed to 1μM crizotinib, 3.3μM ZSTK474, 0.1μM salinomycin or their combination and the plates were visually inspected under microscope weekly. At three months of exposure to the drugs, growing colonies were present only in the crizotinib treated wells when one of three wells were fixed and stained as for colony formation assays. One of the crizotinib resistant wells (H3122CR cells) was trypsinized and the cells were transferred to non-drug containing media. After a single passage cells were 1) analyzed with western blot and MTS-assays, 2) plated on 96-well plates on average one cell per well to generate clones, or 3) let to grow in non-drug media and analyzed with MTS assay for presence of resistance every three passages. Combination treated wells were followed for additional three months. After six months, the experiment was terminated since there were no surviving cells.
